# Milk Osteopontin for Gut, Immunity and Brain Development in Preterm Pigs

**DOI:** 10.3390/nu13082675

**Published:** 2021-07-31

**Authors:** Karoline Aasmul-Olsen, Nicole Lind Henriksen, Duc Ninh Nguyen, Anne Birgitte Heckmann, Thomas Thymann, Per Torp Sangild, Stine Brandt Bering

**Affiliations:** 1 Comparative Pediatrics and Nutrition, Department of Veterinary and Animal Sciences, University of Copenhagen, 1870 Frederiksberg C, Denmark; karolineaas@sund.ku.dk (K.A.-O.); nlh@sund.ku.dk (N.L.H.); dnn@sund.ku.dk (D.N.N.); thomas.thymann@sund.ku.dk (T.T.); pts@sund.ku.dk (P.T.S.); 2 Arla Foods Ingredients Group P/S, Arla Foods Ingredients, 8260 Viby J, Denmark; anne.birgitte.lau.heckmann@arlafoods.com

**Keywords:** osteopontin, bioactive milk protein, preterm neonates, intestinal health, systemic immunity, brain development, cognition

## Abstract

Deficient levels of milk osteopontin (OPN) in infant formula may partly account for developmental differences between infants fed formula or maternal milk. We hypothesized that a milk diet supplemented with bovine milk OPN improves gut, immunity and brain development and tested this in a preterm pig model. Preterm pigs delivered by cesarean section (90% gestation) were fed raw bovine milk (CON, *n* = 19) or the same diet supplemented with a physiologically relevant dose of OPN (46 mg/(kg·d), *n* = 16). Endpoints related to clinical outcomes, systemic immunity and neurocognitive development were assessed during the study and gut tissues were collected at Day 19. Growth pattern, early motor development and most systemic immune parameters were similar between OPN and CON pigs. The OPN pigs had higher villus-to-crypt ratios than CON pigs and higher monocyte and lymphocyte counts on Day 8. Gut digestive and absorptive functions and cognitive performance (T-maze test) were similar between OPN and CON pigs. In conclusion, dietary supplementation with OPN above basal bovine milk levels induced minor improvements in gut structure and systemic immunity without any effects on cognitive performance. The minimal levels of OPN in infant formula to secure optimal adaptation in the immediate neonatal period remain to be determined.

## 1. Introduction

Optimizing bioactive protein levels in infant formulas may help to reduce the well-established differences in development and disease resistance between infants fed formula and maternal milk [1,2,3]. Osteopontin (OPN) is an acidic protein with high glycosylation and phosphorylation that is considered important for stimulating development in the immediate neonatal period and it is abundantly present in early human milk [4,5,6]. The OPN milk concentrations vary remarkably between species, and in human milk even the inter-individual variation between mothers is high. A decline during the lactation period seems though a consistent observation [5,7]. In a multicenter study, the median OPN level was 160 mg/L at about 13 weeks postpartum [7]. The top 25% level of OPN in human milk was 230–495 mg/L, which is much higher than the mean levels reported for bovine milk (~18 mg/L) or infant formulas (~9 mg/L) [4]. Currently, bovine milk OPN is commercially accessible and can hence be added to infant formula to reach levels similar to the levels found in human milk. 

Due to the highly conserved arginine-glycine-aspartate (RDG) integrin binding site in OPN, the biological effects can be expected to be similar across species [8]. The fragment containing the RDG sequence is highly resistant to breakdown due to glycosylations close to the integrin binding motif [9], thus supporting the bioactive potential of native milk OPN in the gut. In vitro, milk OPN is able to stimulate intestinal cell proliferation and exert antibacterial activities as part of a complex with lactoferrin (LF), another important bioactive milk protein [10,11]. The beneficial effects of milk OPN in the gut have also been demonstrated in vivo. The intestinal development in newborn rhesus monkeys has been improved by bovine milk OPN and the jejunal transcriptome of OPN-supplemented formula-fed animals were similar to that in breastfed counterparts [12]. Dietary milk OPN effects, both locally in the gut as well as systemically following minor intestinal absorption in infants, have partly been attributed to its ability to stimulate endogenous OPN levels [5,13,14]. OPN is found in most body tissues and fluids where it exerts a variety of functions such as biomineralization and immune modulation besides participating in cell proliferation, migration, adhesion and opsonization [8,10,15,16]. OPN is known to regulate the Th1/Th2 balance, which in neonates is skewed towards a Th2 polarization [17]. OPN possess cytokine-like properties and can stimulate Th1 type immunity by induction of IL-12 expression and inhibition of the production of IL-10, the Th2 cytokine, in macrophages [18]. In addition, OPN may support brain maturation, potentially contributing to the improved cognitive functions in breastfed versus formula-fed infants [19,20,21]. In support of this, OPN-fed mouse pups showed better learning ability and higher expression levels proteins related to myelination, including myelin basic protein and myelin-associated glycoprotein, and OPN also enhanced myelination in vitro [14,22,23]. In term pigs, several brain regions were affected by increased relative volume following dietary supplementation with bovine milk OPN but no effects on cognition parameters were observed [24]. 

Preterm infants (birth < 37 weeks of gestation) are particularly diet-sensitive due to their relatively immature gut, immune system and brain. Such infants are susceptible to develop both short- and long-term complications such as necrotizing enterocolitis (NEC), feeding intolerance, infections and impaired neurodevelopment [25,26,27,28]. Bovine milk OPN supplementation has previously been shown to reduce NEC severity in formula-fed preterm piglets [29], and other milk factors have proven to support brain structure and cognitive development particularly in preterm newborns [30,31]. On this background, we hypothesized that preterm newborns, fed a standard milk diet with relatively natural bioactivity, would benefit from further OPN supplementation. Following an initial in vitro demonstration of OPN antimicrobial activity, we used diet-sensitive preterm pigs [32,33,34] to investigate if an OPN-enriched bovine milk diet would improve gut, immunity and brain development in the first three weeks after preterm birth.

## 2. Materials and Methods

### 2.1. In Vitro Antibacterial Assay

First, an antibacterial assay [35] was used to assess the bioactivity of OPN by quantifying its ability to inhibit the growth of, or kill, *Staphylococcus epidermidis*, i.e., a common neonatal Gram-positive pathogen [36]. The same intact unpasteurized bovine milk used as the base for the enteral diets in the pig study, was thawed and centrifuged consecutively at 500× *g* and 3000× *g*, 4 °C for 15 min, aspirating the aqueous layer each time to remove the fat content, while minimizing the loss of milk proteins [37]. Following sterility testing of the skimmed bovine milk, the OPN powder (Lacprodan^®^ OPN-10, Arla Foods Ingredients, Viby, Denmark) was dissolved at concentrations of 1.6 (same as the amount added on Day 1 in the pig study to the OPN milk), 2.8, 5, 10 and 20 g/L, creating 6 test diets, including unsupplemented skimmed bovine milk, which were stored in aliquots of 1 mL at −80 °C until analysis. Frozen mid-log culture stocks [35] of *S. epidermidis* (WT1457 [38], kindly provided by Dr. Xiaoyang Wang, University of Gothenburg, Sweden) were thawed, centrifuged at 3200× *g* and resuspended in sterile saline before incubation with the 6 test diets (in addition to a commercially available ready-to-feed preterm milk formula, included as a negative control [1]) at a calculated dose of 10^5^ and 10^6^ CFU/mL for 2, 4, 6 and 24 h at 37 °C. The final inoculation doses and bacterial growth in the test diets and infant formula after different incubation times were determined [35]. Independent triplicates of the antibacterial assay were performed. 

### 2.2. Animals and Experimental Design

**Figure 1:**
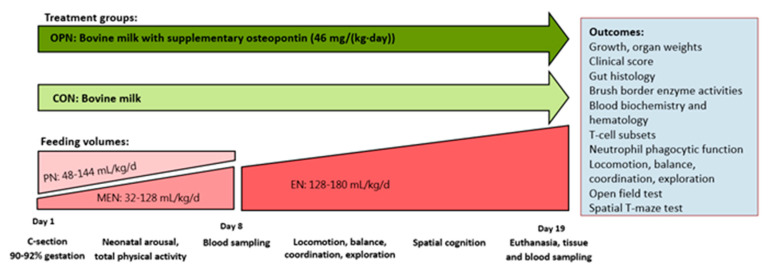
Outline of study design, treatment groups and interventions. OPN, osteopontin; CON, control.

All animal procedures were approved and conducted under a license (license nr.: 2014-15-0201-00418) issued by the Danish Animal Experiment Inspectorate. Forty-eight piglets (Danish Landrace x Yorkshire x Duroc) from two commercially bred sows were delivered preterm by caesarian section at approximately 90% gestation (full term: 117 ± 2 d). Upon successful resuscitation and while still anesthetized from maternal anesthetics, the surgical placement of an indwelling umbilical arterial catheter to facilitate parenteral nutrition (PN) and an orogastric feeding tube to facilitate enteral nutrition (EN) was performed as previously described [32]. Six piglets were euthanized immediately after birth due to failed resuscitation, and were consequently excluded from the study. In total, forty-two piglets were stratified according to sex and birth weight and assigned one of two groups: one group receiving raw bovine milk supplemented with OPN (OPN, *n* = 21) and a control group receiving unsupplemented raw bovine milk (CON, *n* = 21) (Figure 1). Maternal plasma was infused in 3 boluses (total of 25 mL/kg) via the umbilical catheter within the first 24 h to secure passive immunization. Pigs were reared individually throughout the study period of 19 days. From Days 1–5 they were reared in ventilated heated incubators (+ oxygenation during the first 24 h), and on Days 5 and 11 they were relocated to bigger cages. All clinical investigators were blinded to the diet treatments throughout the experimental period. 

### 2.3. Nutrition and Feeding 

The enteral diet fed to all pigs was based on unpasteurized intact bovine milk collected in two batches from a local Jersey cow dairy farm and stored at −20 °C. The bovine milk was fortified with minerals and vitamins (15 g/L Paediatric Seravit, Nutricia, Utrecth, The Netherlands) and electrolytes (6 g/L Revolyte Nutrition, Gunnar Kjems ApS, Copenhagen, Denmark). This milk base served as the complete enteral diet for piglets in the CON group. For pigs in the OPN group, the bovine milk base was further supplemented with bovine milk OPN (Lacprodan^®^ OPN-10) at a median supplemental OPN concentration of 319 mg/L (IQR 272-445) to obtain a daily fixed add-on OPN dose of 46 mg/(kg·day). The concentration is within the human milk range of OPN [4]. The concentration of OPN in the collected bovine milk was 91 mg/L as measured by liquid chromatography-tandem mass spectrometry (LC-MS/MS, Alphalyse A/S, Odense, Denmark). The supplementary dose of OPN was selected to be within the higher end of the human milk range to secure a reasonable difference from the bovine milk control diet. Daily preparations of the enteral diets were made and stored at 4 °C until use (<24 h). 

Pigs received gradually increasing volumes of EN from 32 mL/(kg·day) on Day 1 to 180 mL/(kg·day) on Day 15 and until euthanasia. The enteral diets were given as boluses. During daytime the boluses were given every second hour and during night every third hour until Day 9. Thereafter, the feeding frequency was reduced to every third hour, day and night. The EN was given through the orogastric feeding tube until Day 5, where pigs were taught to drink from troughs. 

The PN was given as a continuous infusion in decreasing amounts from Days 1–8 (144–148 mL/(kg·day)) to counterbalance the small enteral volumes the piglets can cope with. The PN solution was a commercially available human parenteral nutrition product (Kabiven, Fresenius Kabi, Uppsala, Sweden) adjusted to the requirements of piglets as previously described [32]. After removal of the orogastric feeding tube on Day 11 and onwards, all pigs had free access to water.

### 2.4. Clinical Evaluation and Tissue Collection

Throughout the study period, pigs were monitored closely (at least every 3 h) and clinical scores were assigned twice daily as previously described [33]. Upon clinical indication, relevant treatments were initiated, e.g., the administration of intramuscular analgesics (meloxicam: Metacam, 5 mg/mL Boehringer Ingelheim, Ingelheim am Rhein, Germany or butorphanol: Torbugesic Vet, 10 mg/mL, Zoetis, Parsippany, NJ, USA). In cases of lack of response to treatment, pigs were euthanized. Body weights were measured every day, and the average body weight gain expressed as g/(kg.d), was calculated according to the formula: (weight at euthanasia–weight at birth)/(average body weight × lifetime). Two times daily, feces were scored as follows: 1 (firm feces), 2 (soft/pasty feces), 3 (creamy feces) and 4 (diarrhea). An oral electrolyte solution (Revolyte Nutrition) was given in between feedings in cases of diarrhea to avoid dehydration. Prophylactic oral antibiotics (metronidazole: Flagyl, 40 mg/mL, Sanofi-Aventis, Paris, France; amoxicillin, 500 mg, with clavulanic acid, 125 mg: Bioclavid, Sandoz GmbH, Copenhagen, Denmark; gentamicin: Gentocin Vet, 4.35 mg/mL, Scanvet, Fredensborg, Denmark) were given two times a day from Days 8–10, following the removal of the umbilical arterial catheter. The same combination of oral antibiotics was administered therapeutically, upon indication. 

On Day 19, 60 min prior to euthanasia, the pigs were given a last standardized bolus of their respective diets. Pigs were anesthetized with an intramuscular injection of zolazepam/tiletamin (Zoletil 50, 25 mg/mL zolazepam and 25 mg/mL tiletamin, Virbac, Carros, France), ketamine (Ketaminol, 50 mg/mL, MSD Animal Health, Kenilworth, NJ, USA) and butorphanol (Torbugesic Vet), and they were thereafter euthanized with sodium pentobarbital (Euthanimal, 400 mg/mL, Scanvet) by an intracardiac injection. Immediately after euthanasia, internal organs were dissected and weighed, both entire organs and specific organ regions (e.g., full/empty stomach, small intestine, colon, whole brain, brain regions, liver, spleen, kidneys, lungs and heart). The cerebral water fraction was estimated by weighing the left cerebral hemisphere before and after dehydration for 14 days at 50 °C. Each of the five gastrointestinal regions (stomach, proximal small intestine (Prox), middle small intestine (Mid), distal small intestine (Dist) and colon) were evaluated macroscopically and assigned a NEC score [39]. Tissue samples from each gastrointestinal region were collected and snap-frozen in liquid nitrogen or fixed in 4% formaldehyde and stored at −80 °C until further analyses. The study design and outcomes are shown in Figure 1. 

### 2.5. Intestinal Mucosa Structure and Brush Border Enzyme Activities

The mucosa structure in the small intestine was assessed by measuring villus height and crypt depth of histology slices of Prox, Mid and Dist tissues fixed in paraformaldehyde and stained with hematoxylin and eosin as previously described [40]. Immunohistochemical staining for Ki-67 of replicates of Prox, Mid and Dist sections, was used to evaluate the enterocyte proliferation in the small intestinal mucosa as previously described [41]. Four images of each replicate were obtained in a systematical random fashion using a light microscope (Ortho-plane, Leitz, Germany), attached to a camera and a 10× objective lens. Image analysis was performed using Fiji:ImageJ (NIH Image, Bethesda, Maryland) and the total area of Ki-67-positive stained nuclei was calculated as a fraction of the total nuclei area. The activity of 6 brush border enzymes (maltase, sucrase, lactase, aminopeptidase N (ApN), aminopeptidase A (ApA) and dipeptidyl peptidase IV (DPPIV)) was determined in mid tissue homogenates using spectrophotometry [42].

### 2.6. Blood Biochemistry, Hematology and Systemic Immunity 

Blood samples for biochemistry and hematological parameters were analyzed on Day 1 from the umbilical cord during C-section, Day 8 from patent umbilical arterial catheters or alternatively the jugular vein and Day 19 from cardiac puncture during euthanasia. Plasma for biochemistry obtained by a heparinized blood vacutainer (BD Diagnostics, Oxford, UK) followed by centrifugation (2500× *g*, 4 °C for 10 min) was analyzed using the ADVIA 1800 Chemistry System (Siemens Healthcare GmbH, Erlangen, Germany). Full blood for hematology and immune cell counts obtained by an EDTA-coated blood vacutainer (BD Diagnostics), were analyzed using the ADVIA 2120i Hematology System (Siemens Healthcare GmbH). 

Systemic immunity related assays to study blood T-cell subsets, neutrophil phagocytosis function and immune competences by ex vivo blood Toll like receptor (TLR) stimulation were performed on full blood samples collected in a heparinized blood vacutainer (BD Diagnostics) on two days; Day 8 from umbilical arterial catheters or alternatively the jugular vein and Day 19 from the jugular vein. 

Blood T-cell subset characterization was measured as previously described [43]. Briefly, erythrocytes were lysed using 1 X BD FACS Lysing solution (BD Biosciences, Franklin Lakes, NJ, USA) and leukocytes were incubated with fixation/permeabilization buffer (Invitrogen, Carlsbad, California) for 30 min at 4 °C in the dark. Following washing with permeabilization buffer (Invitrogen), cells were blocked with porcine serum (ThermoFisher Scientific, Waltham, MA, USA) for 15 min 4 °C in the dark and subsequently stained with four fluorescently labelled monoclonal antibodies; PE-Cy7- conjugated mouse anti-pig CD3 antibody (clone BB23-8E6-8C8, BD Biosciences), FITC-conjugated mouse anti-pig CD4 antibody (clone MIL17, Bio-Rad Laboratories, Hercules, California), PE-conjugated mouse anti-pig CD8 antibody (clone MIL12, Bio-Rad Laboratories) and APC-conjugated rat anti-mouse FOXP3 antibody (clone FJK-16s, ThermoFisher Scientific). Samples were analyzed using a BD Accuri C6 flow cytometer (BD Biosciences) and populations of T-cells (CD3+ lymphocytes), cytotoxic T-cells (CD3+CD4-CD8+ lymphocytes), helper T-cells (CD3+CD4+CD8- lymphocytes) and regulatory T-cells (CD3+CD4+FoxP3+ lymphocytes) were identified. 

The phagocytic function of blood neutrophils was analyzed by flow cytometry using the pHrodo Red Escherichia coli (560/585 nm) Bioparticles Phagocytosis Kit for Flow Cytometry (ThermoFisher Scientific) [34]. The phagocytic rate was indicated by the percentage of neutrophils exerting phagocytosis in the neutrophil population (pHrodo+) and the phagocytic capacity of the neutrophils was indicated by the median fluorescent intensity (MFI). 

To assess the systemic inflammatory response to an ex vivo Gram-negative bacterial challenge, 294 µL full blood was incubated with a TLR4 agonist (lipopolysaccharide (LPS), E. coli O127:B8, Sigma Aldrich, St. Louis, MI, USA) for 5 h at 37 °C, 5% CO_2_. Stimulated and unstimulated blood was then centrifuged at 2000× *g*, 4 °C for 10 min and plasma supernatants were analyzed for tumor necrosis factor (TNF)-α and Interleukin (IL)-10 by ELISA (Porcine Specific Duoset Kits, R&D Systems, Abingdon, UK), following the manufacturer’s guidelines. The plasma samples were diluted 2–2.5 times prior to analysis. Only samples with cytokine absorbance levels within the standard range were included for the calculation of mean cytokine levels and ratios. However, samples with cytokine absorbance levels below the lowest standard but higher than the blank were given an arbitrary value of half of the lowest detected level in the plate and included in the statistical analysis.

### 2.7. Early Motor Development, Behavior and Cognition 

The time to first stand and first walk after birth was measured as estimates of basic motor skill acquisition. Motion-sensitive infrared cameras installed above each home cage were continuously recording the pigs’ motor activity from Day 1 at 21:00 p.m., when pigs were fully recovered from maternal anesthetics, to Day 11 [33]. The mean proportion of active time during an hour was calculated from recordings of 12 hour intervals during day-time (9:00 a.m. to 9:00 p.m.) and night-time (from 9:00 p.m. to 9:00 a.m.). 

The exploratory behavior of each pig was assessed on Day 9 in an open field arena (1.2 m × 1.2 m) with a video camera recording from a birds view during a 3-min recording period with pigs in randomized order. Pigs were assigned a real-time overall score by two blinded observers based on four objective parameters: floor contact, wall contact, escape attempts and number of gaits. The acquired videos were subsequently analyzed using EthoVision XT10 (Noldus Information Technology, Wageningen, The Netherlands). The outcomes were total distance moved and velocity calculated for each pig. 

Following adequate visual development and a period of habituation, the spatial cognition of each pig was assessed in a randomized order during Days 12–18, using the standardized spatial T-maze cognition test [44]. Hippocampus-dependent learning and memory were tested during 1–2 acquisitions a day, each consisting of 10 trials. Extra-maze visual cues were applied for the pigs to navigate to locate a fixed milk reward. Alternating starting positions of pigs were used to avoid ego-centric response mechanisms and milk was placed in both reward arms. To mask olfactory signals, the milk was though only accessible in one of the arms. The number of correct choices per acquisition were recorded during a standard phase (Acquisitions 1–6, Days 12–17) and a reversal phase (Acquisitions 7–9, Days 17–18). In the reversal phase, the milk reward was changed to the opposite arm. A minimum of 80% correct choices (8 correct choices out of 10 trials) were defined as the learning criterion [30].

### 2.8. Statistical Analysis 

The statistical software R (version 3.6.0) was used for all statistical analyses. For the antibacterial assay, differences in *S. epidermidis* growth among the 6 test diets and infant formula across 2–24 h of incubation, were analyzed using a linear mixed effects model (lme function) with inoculation dose, time and test diet/formula as fixed effects (including interaction between the last two) and the experimental period as a random effect, followed by pairwise comparisons at each time point (lsmeans package). Continuous repeated measurements from the pig study (i.e., body weight, home cage activity, correct choices in the spatial T-maze cognition test and small intestinal parameters measured across several regions) were analyzed using a linear mixed effects model (lme function) including the appropriate correlation structure and pig as a random effect. The lsmeans package facilitated subsequent comparisons within each small intestinal region. A cox proportional hazard model (coxph function) was used for continuous time-to-event data (i.e., the acquisition of basic motor skills and first time presenting with diarrhea). All other continuous data were analyzed using a multiple linear regression model (lm function). The Holm correction was applied to all blood biochemistry data to correct for multiple comparison testing and control the family-wise error rate. Discrete time-to-event data (i.e., the percentage of pigs that reached the T-maze learning criterion) were analyzed using a generalized multiple linear regression model (glm function) with a binomial response and a complementary log-log link. Sex, litter and birth weight were identified as biologically relevant potential confounders prior to the study and thus included as fixed effects in all of the above-mentioned models. Additionally, for all blood biochemistry and hematological parameters, the baseline levels measured on Day 1 prior to group randomization were included as fixed effects in the corresponding multiple linear regression models. 

Model validation of linear regression models were performed by graphical displays of the normality and homoscedasticity of the residuals and fitted values, and data transformations (log or sqrt) to satisfy these assumptions, were performed if needed. For variables that could not conform to normal distribution, the nonparametric Mann–Whitney test was applied. Regression models with categorical response variables were validated by investigating the cumulated residuals using the goodness-of-fit test (gof package). Unless otherwise stated, raw data are presented as means ± SD and *p*-values < 0.05 are considered statistically significant.

## 3. Results

### 3.1. In Vitro Antibacterial Assay

**Figure 2:**
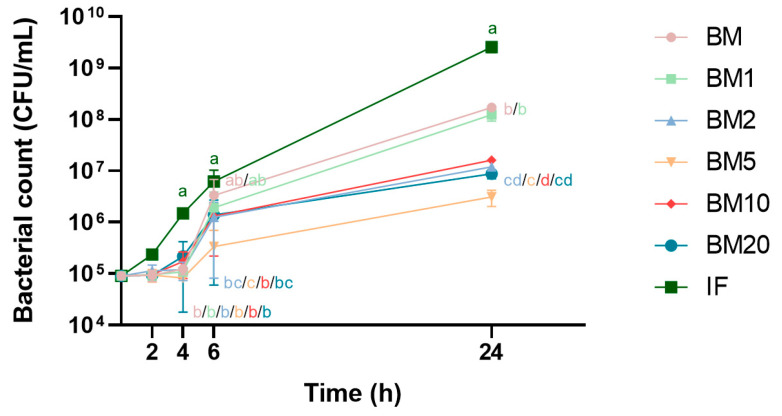
Growth curves of *S. epidermidis,* inoculated at a dose of 10^5^ after incubation with infant formula (IF) or unpasteurized skimmed bovine milk (BM) with differently added concentrations of bovine milk osteopontin (BM1, 1.6 g OPN/L; BM2, 2.6 g OPN/L; BM5, 5 g OPN/L; BM10, 10 g OPN/L; BM20, 20 g OPN/L). Values are expressed as mean ± SD (*n* = 3). Each incubation time point not sharing the same letters are significantly different (*p* < 0.05).

The effects of skimmed bovine milk supplemented with OPN at different concentrations, on the growth and viability of *S. epidermidis*, were evaluated and compared to infant formula. After 4, 6 and 24 h of incubation with infant formula, the *S. epidermidis* concentration increased by approximately 10, 50 and 1 × 10^4^-fold, respectively, for both inoculation doses (10^5^ and 10^6^ CFU). There were no differences between the 6 test diets and formula before 4 hours of incubation with the inoculation dose of 10^5^ CFU (Figure 2). However, an apparent bactericidal effect of the bovine milk-based test diets was evident compared to infant formula, as the *S. epidermidis* concentration remained static in these during 1–4 h of incubation. At 4 h of incubation, following the same inoculation dose, bacterial counts were lower in all 6 test diets compared to formula (approximately 10 times, *p* < 0.01). The OPN supplementation exerted dose-dependent long-term bacteriostatic effects against *S. epidermidis* as bacterial counts after 6 h of incubation were significantly lower in the test diets containing the 4 highest OPN doses (*p* < 0.05, 10^5^ inoculation dose). Only unsupplemented skimmed bovine milk lost momentarily its capacity to prevent growth of bacteria when incubated for 6 hours (10^5^ CFU inoculation dose). At 24 h of incubation, bacterial counts were lower in all 6 test diets compared to formula (*p* < 0.001, 10^5^ CFU inoculation dose). All 6 test diets exerted bacterial inhibitory effects (initially bactericidal) against *S. epidermidis*, inoculated at 10^6^ CFU, at all tested time points (<2 h of incubation *p* < 0.07, >2 h of incubation *p* < 0.05).

### 3.2. Clinical and Growth Outcomes 

**Figure 3:**
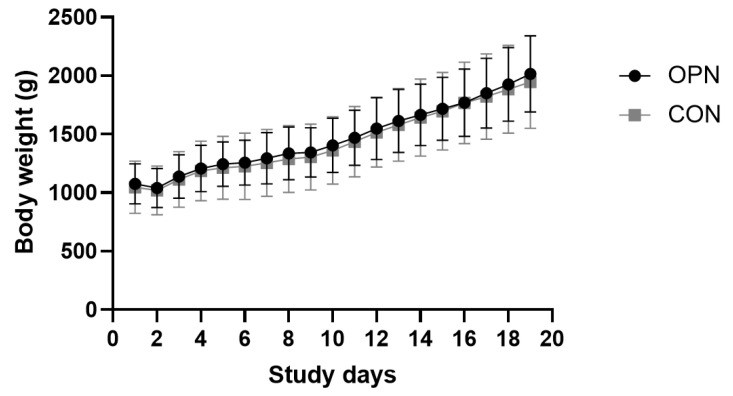
Growth curves based on daily body weights of the preterm pigs from Days 1–19. The pigs received either a bovine milk diet (CON, *n* = 19) or the milk diet supplemented with bovine milk osteopontin (OPN, *n* = 16). Data are expressed as mean ± SD.

Seven pigs (5/21 OPN compared with 2/21 CON, *p* = 0.4) out of the 42 pigs were euthanized/died as a consequence of immaturity related/iatrogenic complications in the beginning of the experiment (<Day 9), including respiratory distress shortly after birth, peritonitis, signs of sepsis, thrombosis or NEC. Unrelated to the diet, these complications did not affect the average life span of the two groups. On Day 19, the post-mortem score of the gastrointestinal tract revealed one isolated small intestinal NEC case in each group (score ≥ 3 out of 6). In both instances, the pigs did not display any clinical signs prior to euthanasia. All pigs were throughout the study period assigned low daily clinical scores of 1–2. All pigs surviving until Day 19 did at some point during the study period develop diarrhea. Time of first presentation with diarrhea was comparable between the two treatment groups, however, different between the two litters (*p* < 0.001). One litter received the intended prophylactic antibiotic treatment on Day 8, whereas for the other litter, a therapeutic antibiotic treatment was initiated on Day 5. The two groups displayed similar growth patterns with an average daily body weight gain of 33.6 ± 4.0 g/(kg·day) and 32.9 ± 3.7 g/(kg·day) for the OPN and CON, respectively (Figure 3). There were no differences between the two groups in birth weights, weights at euthanasia or any of the relative organ dimensions (Table 1).

### 3.3. Gut Structure and Function

**Figure 4:**
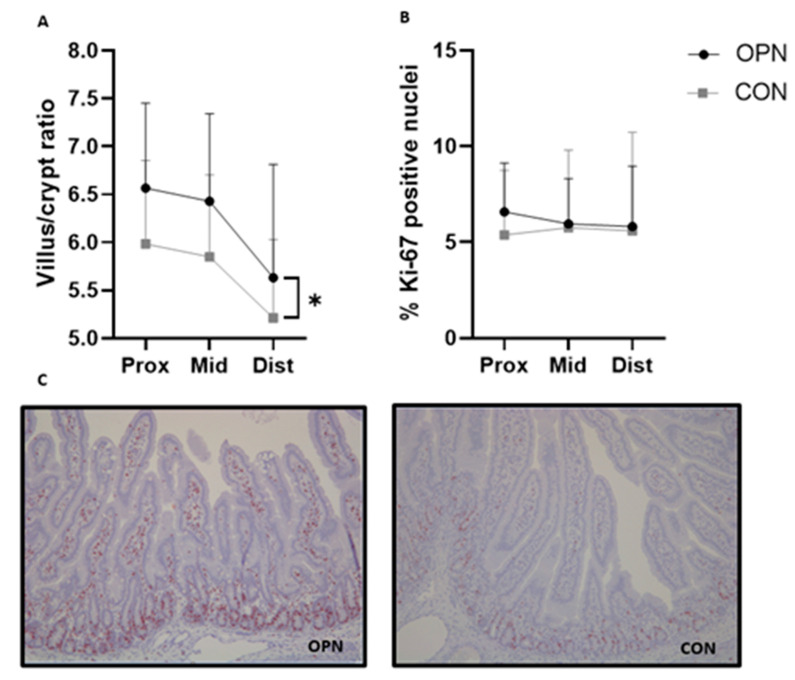
Villus-to-crypt ratio (**A**) and % Ki-67 positive nuclei (**B**) in the small intestine of preterm pigs. The pigs received a bovine milk diet (CON, *n* = 19) or the milk diet supplemented with bovine milk osteopontin (OPN, *n* = 16). Immunohistochemical staining for Ki-67 in OPN and CON proximal small intestinal tissue (**C**). Data are expressed as mean ± SD. * *p* < 0.05. Dist, distal small intestine; Mid, middle small intestine; Prox, proximal small intestine.

Villus height and crypt depth, assessed in the proximal, middle and distal part of the small intestine were similar between the two groups (Table 2). Compared to CON, an overall higher villus-to-crypt ratio across the three small intestinal regions was measured in OPN (*p* < 0.05, Figure 4A). Similar amounts of proliferative enterocytes were observed between the two groups, indicated by the same relative area of Ki-67 positive stained nuclei in the three small intestinal regions (Figure 4B,C). Further, the gastric emptying ability indicated by the gastric content weights at euthanasia as well as the activity of lactase, maltase, sucrase, ApA, ApN and DPPIV measured in the middle part of the small intestine were comparable between the groups (Table 2).

### 3.4. Blood Biochemistry, Hematology and Systemic Immunity 

**Figure 5:**
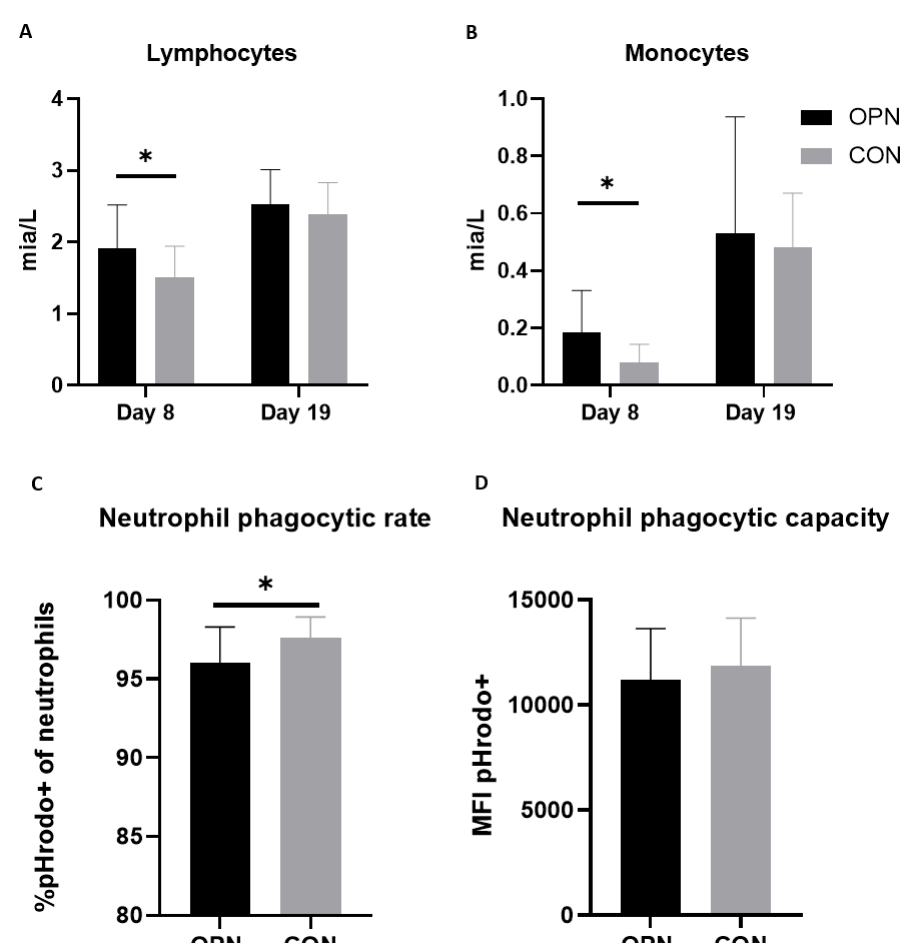
Full blood lymphocyte (**A**) and monocyte (**B**) counts in preterm pigs on Days 8 and 19. The pigs received a bovine milk diet (CON, *n* = 12–18) or the milk diet supplemented with bovine milk osteopontin (OPN, *n* = 9–14). Neutrophil phagocytic rate (**C**) and capacity (**D**) on Day 19 of OPN and CON pigs. Data are expressed as mean ± SD. * *p* < 0.05. MFI, mean fluorescent intensity.

Cord blood samples revealed a higher total leucocyte and basophil count on Day 1 (*p* < 0.05) of pigs later assigned the OPN group relative to pigs assigned the CON group. No other immune cell counts, blood biochemistry or hematology variables differed between the two groups prior to receiving their specific dietary interventions (data not shown). Blood biochemistry variables measured on Day 8 and 19 did not differ between groups (Table S1, Supplementary Materials), and accompanied hematology parameters and immune cell counts are presented in Table 3. Compared with CON pigs, OPN pigs had a higher lymphocyte and monocyte count on Day 8 (*p* < 0.05, Figure 5A,B). The lymphocyte and monocyte counts increased from Day 8 to Day 19, with no final differences between the groups (Figure 5A,B). The remaining immune cell counts and nonimmune-related hematological parameters were similar between OPN and CON pigs (Table 3).

Innate and adaptive immune parameters were evaluated on Days 8 and 19. On Day 8, the blood neutrophil phagocytic rate and capacity were similar between OPN and CON (Table 3). Conversely, on Day 19 the CON had a higher frequency of neutrophils exerting phagocytosis compared with OPN (*p* < 0.05). The phagocytic capacity was though not different between groups (Figure 5C,D). The T-cell subset characterization on Days 8 and 19 did not reveal any differences between T helper cells, cytotoxic T cells and regulatory T cells in the two groups (Table 3). A representative FACS gating is shown in Figure S1, Supplementary Materials.

The TLR4-mediated IL10 and TNF-α cytokine production of full blood from Days 8 and 19 was evaluated. TNF-α and IL10 were detected at similar levels between OPN and CON in unstimulated plasma obtained on Day 8 and there were no group differences in the response to LPS stimulation indicated by a similar TNF-α/IL10 ratio (Table S2, Supplementary Materials). TNF-α was not detectable in unstimulated plasma obtained on Day 19 from either group. However, both groups responded equally to LPS stimulation, indicated by similar TNF-α and IL-10 plasma levels (Table S2, Supplementary Materials). 

### 3.5. Brain Structural and Functional Outcomes 

**Figure 6:**
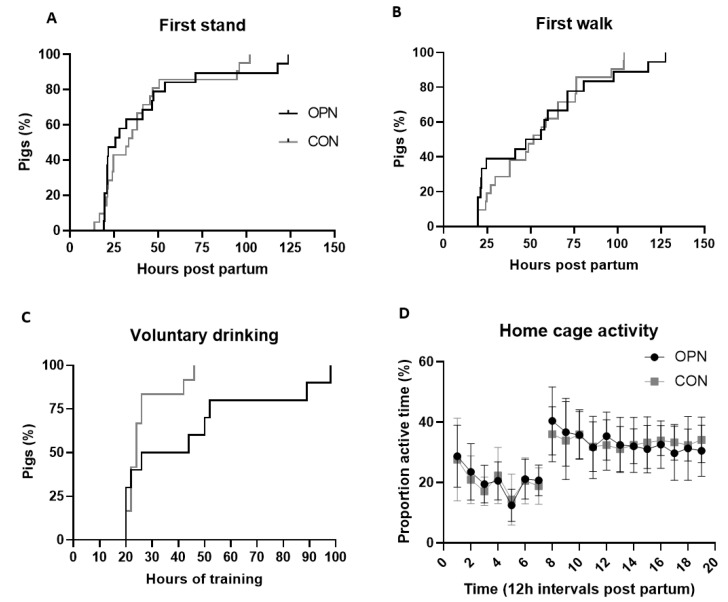
Early motor development, indicated by the time to first stand (**A**), first walk (**B**), first voluntary intake of a full milk bolus (**C**) and the proportion of active time during an hour averaged over 12 h intervals from Days 1–11 (**D**), of preterm pigs fed a bovine milk diet (CON, *n* = 12–21) supplemented with bovine milk osteopontin (OPN, *n* = 10–19). Data are expressed as mean ± SD. pp, postpartum.

Structural brain related outcomes including the absolute and relative values of the total brain weight, regional brain weights and the cerebral water content were similar between the OPN and CON group (Table 4). No differences in early motor development were observed, as time to first stand, first walk and first voluntary intake of a full milk bolus as well as the proportion of active time in home cages, measured by motion-sensitive cameras from Days 1–11, were similar between OPN and CON (Figure 6A–D). 

**Figure 7:**
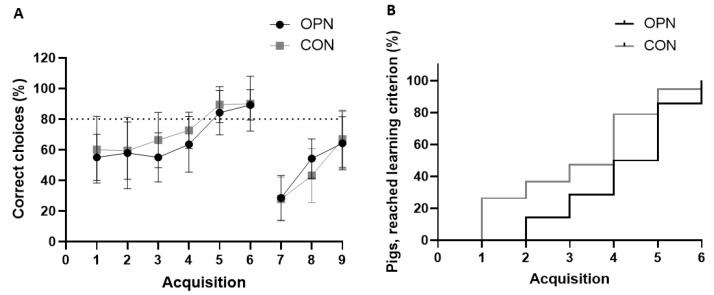
Cognitive performance during the spatial T-maze test. Proportion of correct choices (**A**) during the standard (Acquisitions 1–6) and reversal phase (Acquisitions 7–9) of preterm pigs fed a bovine milk diet (CON, *n* = 19) supplemented with bovine milk osteopontin (OPN, *n* = 14). The proportion of OPN and CON pigs reaching the learning criterion of 80% correct choices in the standard phase (**B**). Data are expressed as mean ± SD.

The pigs’ exploratory behavior evaluated in the open field arena on Day 9 were comparable between the two groups, as the overall score, total distance moved and velocity were similar (Table 4). Further, a similar cognitive performance was observed during the standard and reversal phase in the spatial T-maze cognition test performed over 9 acquisitions from Day 12–18 (Figure 7A,B). 

The mean proportion of correct choices over time increased for both groups in both phases (Figure 7A). In the standard phase, both OPN and CON reached the predefined learning criterion on Acquisition 5 (80% correct choices, Figure 7A). All pigs reached the learning criterion, and there were no difference in the time taken to reach the learning criterion (Figure 7B).

## 4. Discussion

The multifunctional effects of OPN, particularly in terms of its proliferative, immune regulatory and anti-bacterial activity, but potentially also in supporting brain development, may help to improve the health and organ development of sensitive neonates and infants. The high OPN levels in early human milk and corresponding infant OPN plasma concentrations advocates sufficient supply of OPN in the immediate neonatal period when infants are fed human milk [5]. In contrary, the lower concentration of OPN in bovine milk and infant formulas [4] provides a window of opportunity for increasing the OPN content of milk formulas, and supplementation with OPN may help to reduce the developmental differences that exist between formula-fed and maternal milk-fed infants [2,3]. In this study, we showed that supplementation of OPN to a bovine milk diet with basal OPN content was well-tolerated, but only minor improvements in gut structure and systemic immunity were observed, and no further effects on brain structure and function were observed. The bovine milk base was selected to secure a relevant bioactive base diet in comparison to a milk formula, which on top of the lower OPN content also has diminished bioactivity.

Milk OPN is recognized as an important biologically active whey protein, mainly due to its ability to modulate immunity and stimulate cell proliferation [18,45]. The ability of OPN to form complexes with the positively charged lactoferrin found in milk may add further functional properties in contributing to the defense against pathogens in the infant gut [10]. The OPN-lactoferrin complex is found in human and bovine milk, but can also be formed from the individual proteins [10]. To document the bioactive potential of OPN in a bovine milk based diet, we initially tested the in vitro antibacterial ability of bovine milk OPN added in different concentrations to sterile skimmed Jersey milk. Our results show that the OPN supplemented milk diets exert both short- and long-term antibacterial ability in a dose-dependent manner by inhibiting the growth of *S. epidermidis*, a common neonatal pathogen causing late-onset sepsis following gut translocation [46,47]. This provides an opportunity to stimulate gut development by OPN supplementation of the neonatal milk diet. 

Optimum dietary OPN levels in milk-based diets are unclear, and the variability of OPN content in human milk is large, both between mothers, countries and over the period of lactation [7]. Previous animal studies have predominantly investigated effects of adding OPN to a control diet without OPN, in levels comparable to the mean human milk content [14,22,24]. In this preclinical pig study, we focused on the clinical relevant aspect of adding more OPN to a diet with a basal OPN level to reach high human milk levels. The OPN supplementation in this study increased the daily dose of OPN to a dose representing the intake in infants from mothers with relative high OPN milk concentration, and well beyond what is normally received from infant formula [4]. The OPN content in the collected Jersey milk turned out to have a higher content of OPN than previously measured in bovine milk. It is of relevance to note that the LC-MS/MS method used is different from the ELISA method used in previous studies [48]. The lack of cognitive effects in the current study may lie in the fact that the control pigs already receive OPN in concentrations of 91 mg/L, as inherently present in the Jersey milk, and the additional OPN supplementation gives no additional benefits. We found that supplementing bovine milk with OPN was well tolerated and conferred minor effects on gut and immune maturation, whereas the brain structural and functional parameters were unchanged. Both the OPN and CON pigs displayed growth rates comparable to or higher than what is normally observed in preterm piglets in similar studies with bovine milk diets [49,50,51]. The growth rates and relatively good clinical status with low NEC score achieved in these preterm pigs, provided optimal conditions to document dietary effects on changes in organ functions and brain cognitive development in sensitive newborns. 

There is a high similarity between human, cow and pig OPN sequences [52], and the ubiquitous nature of OPN is well established in pigs. OPN is expressed by a variety of healthy porcine tissues with the greatest abundance found in ileal tissue [53], but porcine milk concentrations are unknown. We have previously shown minor beneficial gut effects of an OPN-enriched formula in preterm piglets in relation to recovery from prenatal inflammation [43]. Al together, this emphasizes the use of the piglet as a preclinical model to investigate effects of dietary OPN supplementation. 

Relative to CON, OPN pigs had a higher villus-to-crypt ratio across the small intestine. A similar morphological difference was reported based on duodenal samples from 10 day old mice pups nursed by wild type dams or OPN knock-out dams supplemented with bovine milk OPN relative to mice pups not receiving any OPN [22]. Previous preterm pig studies have correlated increased villus heights with an enhanced nutrient absorptive capacity of, e.g., galactose [51,54], which may be critical to secure optimal growth. Using the porcine IPEC-J2 cell line we have previously demonstrated in vitro the ability of OPN to increase cell proliferation in a dose-dependent manner [43]. The improved gross intestinal mucosal structure observed among OPN pigs in the current study could not be explained by an increase in proliferative enterocytes based on the Ki-67 staining in the small intestine, and did not result in changed digestive enzyme activities. Earlier findings in OPN supplemented formula-fed newborn rhesus monkeys, have shown intestinal changes with jejunal gene expression profiles to be more similar to breastfed counterparts [12]. 

The same developmental trajectory regarding immune cell counts was observed in OPN and CON pigs. All immune cells, except basophil and eosinophil counts, with similarly low levels in term piglets [34], increased in number from Day 8 to Day 19, indicating an enhanced capability of both groups to mount a sufficient immune response to a potential pathogen. The circulating monocyte and lymphocyte levels of OPN pigs on Day 8 were higher relative to CON pigs but not at Day 19. Since the clinical condition was similar between pigs in both groups and NEC scores low, the monocyte and lymphocyte levels are probably mainly related to treatment effects and suggests an accelerated immune development in OPN pigs. A higher proportion of circulating monocytes was also observed in a randomized controlled trial of six-month-old infants receiving formulas with added bovine milk OPN at concentrations of 65 mg/L and 135 mg/L, relative to breastfed controls [55]. OPN is able to interacts with immune cells via its receptor, and act as a chemotactic agent for IL-1-activated monocytes, which may be physiologically relevant during inflammation [16]. OPN may further play a role in innate immunity by mediating phagocytosis through the integrin receptor, α_X_β_2_, expressed on the surface of monocytes [16]. Further studies investigating the role of OPN on monocyte function and how this translates into benefit for formula fed infants remain to be investigated.

A T-cell subset characterization was performed on Days 8 and 19 to further describe the lymphocyte population, but did not reveal any differences in subset fractions between the two groups in this immediate neonatal period. Infants that have received infant formula supplemented with OPN (65 or 130 mg/L), have shown increased proportion of T-cells in the first six months of life compared with those fed basal formula [55]. This response may not be developed already within the first weeks of life, or the relatively high level of OPN in our base bovine milk (91 mg/L) may limit further changes. The number of T-cells, helper T-cells and cytotoxic T-cells did not differ between OPN and CON, and therefore cannot explain the differences in circulating lymphocytes between the two groups on Day 8. In this study, we stained for CD3, CD4, CD8 and Foxp3, and it remains to be investigated if the higher lymphocyte count could be originating from a higher B-cell count or an increased number of natural killer cells. Systemic immunity maturation after birth includes a shift in the Th1/Th2 balance, where the defense against bacterial infections demands a Th1 skewing [17]. OPN is a potent chemoattractant with cytokine like properties and is known to regulate the Th1/Th2 balance [18]. In this study, the Th1/Th2 balance shortly after birth was not modulated by diet indicated by a similar TNF-α/IL10 ratio on Day 8. As TNF-α levels were undetected in unstimulated plasma samples from Day 19, the TNF-α/IL10 ratio at this point could not be calculated. 

Recently, studies using animal models [14,22,24] have investigated the role of milk OPN in neurodevelopment during infancy. Using an OPN knockout mouse model, milk with OPN levels similar to human milk has been shown to improve development of motor skills and hippocampus-dependent memory abilities in 30-day-old mouse pups compared to dietary OPN-deficient controls. These functional brain outcomes of dietary milk related OPN were linked to enhanced endogenous brain OPN levels and a higher proliferation and differentiation of nerve/glial antigen two cells into oligodendrocytes leading to an increased expression of myelin-related proteins. In a similar experimental setup, wild type mouse pups nursed by OPN knockout dams and given supplemental bovine milk OPN (12 µg/g) exhibited a greater memory ability than unsupplemented controls [22]. Collectively, these results indicate that milk OPN plays an important role in neurocognitive development and that bovine milk OPN may exert such beneficial effects across species. We found similar development in the acquisition of basic motor skills, home-cage activity and exploratory behavior in the open field arena between groups. Group performances in the spatial T-maze test further indicated similar hippocampus-dependent learning and memory and thereby cognitive abilities. Our findings are in accordance with a recently conducted study that compared two-day-old term piglets receiving a soy protein-based formula diet containing no OPN or supplemented with 250 mg/L of bovine milk OPN [24]. Only limited behavioral differences were evident between the two groups assessed during a novel object recognition task performed on postnatal Day 30. This implies that at least in healthy, term born pigs, OPN milk supplementation does not further improve brain-related development as measured by novel object recognition. Similar to the spatial T-maze test [44], the novel object recognition task is considered hippocampus-dependent and therefore these behavioral tests might be more sensitive closer to the time of maximum hippocampal growth rates, which in term male and female pigs occur at approximately postnatal Weeks 8 and 3, respectively [56]. It is not known if long-term cognitive improvements following milk OPN supplementation in early life, as demonstrated in the mouse-model studies [14,22], could have been documented in the pigs at later time points. 

In regard to structural brain development, it was previously documented through magnetic resonance imaging that dietary OPN supplementation increased a number of brain regional volumes following normalization to the total brain volumes [24]. In this study, we found no diet-induced differences in brain regional weights, neither absolute weights nor relative to the total brain weights. We, therefore, did not perform more in-depth analysis such as measuring the gene expression levels of myelin-related proteins, e.g., myelin basic protein and myelin associated glycoprotein [14,23]. It has previously been documented that exogenous OPN has neuroprotective properties by attenuating intracerebral hemorrhage-induced neuroinflammation leading to improved neurological scores [57]. Further research is warranted to investigate if milk OPN is able to confer beneficial structural and functional effects to the brain during systemic inflammatory conditions, often experienced by preterm infants [26]. This was out of the scope for the current study. 

## 5. Conclusions

In a preterm pig model of sensitive newborn infants, we showed that a bioactive bovine milk diet supplemented with additional bovine milk OPN is well-tolerated and induced minor improvements in gut structure and systemic immunity, with no further effects on brain structural or functional development. Supplementation with OPN above what is received from basal bovine milk, thereby reaching levels in the high physiological end of what infants naturally receive from human milk, may therefore have limited effects on gut, immunity and brain development in sensitive newborns in the immediate neonatal period. More information on benefits of milk OPN supplementation of bovine milk based infant formulas to stimulate development of compromised newborn infants remains to be determined. 

## Figures and Tables

**Table 1:** Growth and organ dimensions of preterm pigs fed a bovine milk diet (CON, *n* = 19–21) or the milk diet supplemented with OPN (OPN, *n* = 16–21).

Parameter	Unit	OPN	CON	*p*-Value
Birth weight	g	1051 ± 46.6	1035 ± 49.0	NS
Weight at euthanasia, Day 19	g	1789 ± 115	1861 ± 101	NS
Relative organ dimensions ^1^				
Small intestine length	cm/kg	279 ± 10.4	287 ± 8.19	NS
Prox weight	g/kg	14.2 ± 0.28	14.1 ± 0.39	NS
Mid weight	g/kg	12.4 ± 0.33	12.3 ± 0.36	NS
Dist weight	g/kg	12.3 ± 0.29	12.1 ± 0.34	NS
Stomach weight	g/kg	6.24 ± 0.18	5.94 ± 0.15	<0.01
Colon weight	g/kg	32.1 ± 2.38	35.7 ± 3.63	NS
Liver weight	g/kg	25.9 ± 0.96	26.3 ± 0.49	NS
Spleen weight	g/kg	3.22 ± 0.18	3.50 ± 0.13	NS
Heart weight	g/kg	7.76 ± 0.30	8.14 ± 0.23	NS
Lungs weight	g/kg	18.6 ± 1.03	21.3 ± 0.96	NS
Kidney weight	g/kg	7.33 ± 0.20	7.09 ± 0.19	NS

^1^ Values are expressed as mean ± SEM. Organ dimensions are normalized to the body weight at Day 19. NS: Not significant. Dist, distal small intestine; Mid, middle small intestine; OPN, bovine milk osteopontin; Prox, proximal small intestine.

**Table 2:** Gut structure and function on day 19 of preterm pigs fed a bovine milk diet (CON, *n* = 19) or the milk diet supplemented with OPN (OPN, *n* = 16).

Parameter	Unit	OPN	CON	*p*-Value
Prox, villus height	µm	604 ± 20.4	576 ± 18.1	NS
Prox, crypt depth	µm	92.7 ± 2.74	96.8 ± 1.89	<0.01
Mid, villus height	µm	590 ± 22.7	546 ± 17.6	NS
Mid, crypt depth	µm	92.2 ± 2.50	93.9 ± 2.16	NS
Dist, villus height	µm	490 ± 19.2	457 ± 14.5	NS
Dist, crypt depth	µm	88.5 ± 2.84	88.4 ± 1.82	NS
Gastric content ^1^	g/kg	17.3 ± 1.90	17.1 ± 0.96	NS
Brush border enzyme activities (Mid)				
Lactase	U/g tissue	34.7 ± 3.15	43.1 ± 3.98	0.08
Maltase	U/g tissue	7.80 ± 1.83	7.31 ± 1.06	NS
Sucrase	U/g tissue	1.80 ± 0.29	1.79 ± 0.18	NS
ApA	U/g tissue	7.00 ± 0.50	6.35 ± 0.44	NS
ApN	U/g tissue	11.2 ± 0.44	10.2 ± 0.60	NS
DPPIV	U/g tissue	3.65 ± 0.27	3.40 ± 0.20	NS

^1^ The gastric content is normalized to the bodyweight. NS: not significant. ApA, aminopeptidase A; ApN, aminopeptidase N; Dist, distal small intestine; DPPIV, dipeptidyl peptidase IV; Mid, middle small intestine; OPN, bovine milk osteopontin; Prox, proximal small intestine. Values are expressed as mean ± SEM.

**Table 3:** Hematological and systemic immunity parameters of preterm pigs on Days 8 and 19. The pigs received a bovine milk diet (CON, *n* = 12-18) or the milk diet supplemented with OPN (OPN, *n* = 9–14).

Parameter	Unit	Day	OPN	CON	*p*-Value
Hematology					
Total erythrocytes	10^12^/L	8 19	3.25 ± 0.18 3.56 ± 0.15	3.20 ± 0.09 3.52 ± 0.11	NS NS
Hemoglobin	mmol/L	8 19	4.32 ± 0.23 4.12 ± 0.15	4.22 ± 0.11 3.98 ± 0.12	NS NS
Hematocrit	%	8 19	22.9 ± 0.01 21.5 ± 0.01	22.3 ± 0.01 21.0 ± 0.01	NS NS
Thrombocytes	10^9^/L	8 19	369 ± 40.9 432 ± 79.2	351 ± 27.3 545 ± 26.0	NS NS
Immune cell counts					
Total leukocytes	10^9^/L	8 19	5.38 ± 0.59 14.2 ± 2.12	4.69 ± 0.31 14.1 ± 1.76	NS NS
Lymphocytes	10^9^/L	8 19	1.91 ± 0.16 2.52 ± 0.16	1.51 ± 0.10 2.39 ± 0.13	0.01 * NS
Monocytes	10^9^/L	8 19	0.18 ± 0.04 0.53 ± 0.14	0.08 ± 0.01 0.48 ± 0.05	0.02 * NS
Neutrophils	10^9^/L	8 19	3.13 ± 0.57 10.9 ± 1.96	3.01 ± 0.31 11.0 ± 1.71	NS NS
Eosinophils	10^9^/L	8 19	0.08 ± 0.04 0.04 ± 0.01	0.03 ± 0.01 0.04 ± 0.01	NS NS
Basophils	10^9^/L	8 19	0.01 ± 0.00 0.01 ± 0.00	0.01 ± 0.00 0.01 ± 0.01	NS NS
Large unstained cells	10^9^/L	8 19	0.06 ± 0.01 0.25 ± 0.08	0.06 ± 0.01 0.17 ± 0.03	NS NS
T-cell subsets					
T-cell frequency (CD3+ lymphocytes)	%	8 19	59.5 ± 2.34 65.4 ± 2.09	59.9 ± 1.96 65.2 ± 1.62	NS NS
T_h_-cell frequency (CD3+CD4+CD8-)	%	8 19	55.0 ± 0.02 49.4 ± 3.08	58.3 ± 0.01 53.2 ± 1.97	0.09 NS
T_c_-cells frequency (CD3+CD4-CD8+)	%	8 19	8.38 ± 0.01 10.1 ± 0.94	8.71 ± 0.01 11.7 ± 0.60	NS NS
T_reg_-cells frequency (CD3+CD4+Foxp3+)	%	8 19	6.82 ± 0.00 4.86 ± 0.30	7.66 ± 0.00 4.94 ± 0.40	NS NS
Neutrophil phagocytic function					
Neutrophil phagocytic rate	%	8	92.4 ± 1.51	91.7 ± 1.58	NS
Neutrophil phagocytic capacity, MFI		8	15,488 ± 1304	15,440 ± 802	NS

Values are expressed as mean ± SEM. * *p* < 0.05. NS: not significant. MFI, mean fluorescent intensity; OPN, bovine milk osteopontin; T_c_, cytotoxic T-cell; T_h_, helper T-cell; T_reg_, regulatory T-cell.

**Table 4:** Structural and functional brain related outcomes of preterm pigs. The pigs received a bovine milk diet (CON, *n* = 19) or the milk diet supplemented with OPN (OPN, *n* = 15–16).

Parameter	Unit	OPN	CON	*p*-Value
Absolute values				
Total brain weight	g	37.0 ± 0.58	37.3 ± 0.49	NS
Cerebrum weight	g	29.2 ± 0.47	29.6 ± 0.48	NS
Cerebellum weight	g	3.81 ± 0.07	3.88 ± 0.05	NS
Brain stem weight	g	3.54 ± 0.06	3.62 ± 0.04	NS
Left hippocampus weight	g	0.65 ± 0.03	0.67 ± 0.02	NS
Left caudate nucleus weight	g	0.33 ± 0.01	0.35 ± 0.01	NS
Cerebral water fraction	%	82.7 ± 0.00	82.9 ± 0.00	NS
Relative values				
Total brain weight	g/kg	18.7 ± 0.91	20.0 ± 1.05	NS
Cerebrum weight	%	79.3 ± 0.17	79.4 ± 0.51	NS
Cerebellum weight	%	10.4 ± 0.13	10.4 ± 0.13	NS
Brain stem weight	%	9.65 ± 0.12	9.71 ± 0.09	NS
Left hippocampus weight	%	1.76 ± 0.07	1.78 ± 0.04	NS
Left caudate nucleus weight	%	0.91 ± 0.03	0.95 ± 0.03	NS
Open field				
Overall score	point	5.75 ± 0.23	5.79 ± 0.21	NS
Total distance moved	m	17.1 ± 2.06	17.6 ± 1.34	NS
Velocity	cm/sec	11.4 ± 1.38	11.7 ± 0.90	NS

Data are expressed as mean ± SEM. For relative values the total brain weight is normalized to the bodyweight at Day 19, whereas the remaining brain regions are normalized to the Day 19 total brain weight and expressed as percentages. NS: not significant. OPN, bovine milk osteopontin.

## Data Availability

The data presented in this study are available on request from the corresponding author.

## Supplementary Materials

The following are available online at https://www.mdpi.com/article/10.3390/nu13082675/s1, Table S1: blood biochemistry variables on Days 8 and 19 of preterm pigs fed a bovine milk diet (CON, *n* = 17–19) supplemented with OPN (OPN, *n* = 13–15), Table S2: full blood cytokine levels before and after LPS stimulation on Day 8 and Day 19 of preterm pigs fed a bovine milk diet (CON, *n* = 4–18) supplemented with OPN (OPN, *n* = 5–14). Figure S1. FACS gating strategy. Dot plots and histograms from the lymphocyte population (P1) were used to identify T cell subpopulation, including T cells (CD3+ lymphocytes), Helper T cells (CD3+CD4+CD8- lymphocytes), cytotoxic T cells (CD3+CD4-CD8+ lymphocytes) and regulatory T cells (Treg, CD3+CD4+Foxp3+ lymphocytes).
