# Supplementary material for: Milk Osteopontin for Gut, Immunity and Brain Development in Preterm Pigs
Source: Nutrients. 2021 Jul 31;13(8):2675. doi: 10.3390/nu13082675 (PMC8400468; doi:10.3390/nu13082675)
Supplement: Supplementary file 1 [file nutrients-13-02675-s001.zip › nutrients-1300536-supplementary.pdf]

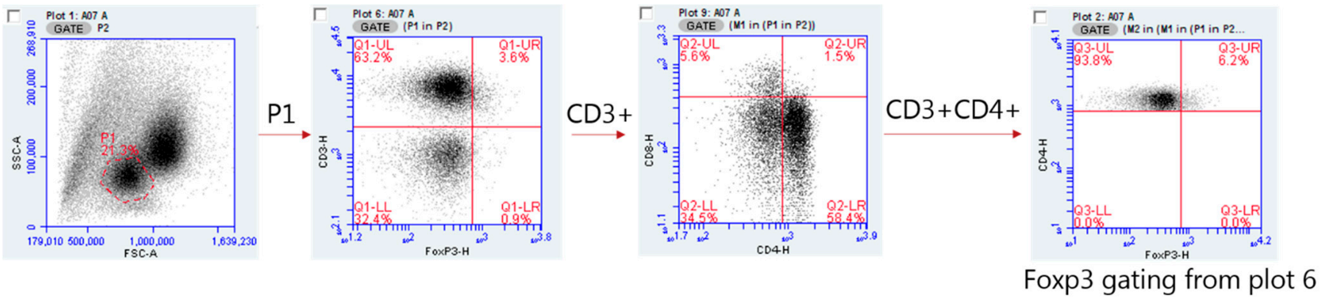

Supplementary Figure S1. FACS gating strategy. Dot plots and histograms from the lymphocyte population (P1) were used to identify T cell subpopulation, including T cells ($CD3^+$ lymphocytes), Helper T cells ($CD3^+CD4^+CD8^-$ lymphocytes), cytotoxic T cells ($CD3^+CD4^-CD8^+$ lymphocytes) and regulatory T cells (Treg, $CD3^+CD4^+Foxp3^+$ lymphocytes).

Supplementary Table S1: Blood biochemistry variables on day 8 and 19 of preterm pigs fed a bovine milk diet (CON, n = 17-19) supplemented with OPN (OPN, n = 13-15).

Parameter	Unit	Day	OPN	CON	Adj. P-Value
Albumin	g/L	8	14.0 ± 0.52	13.6 ± 0.63	NS
		19	16.4 ± 0.86	16.4 ± 0.59	NS
Globulin	g/L	8	19.0 ± 0.39	18.1 ± 0.53	NS
		19	15.4 ± 0.66	15.2 ± 0.34	NS
Albumin/globulin ratio		8	0.74 ± 0.02	0.72 ± 0.02	NS
		19	1.07 ± 0.03	1.09 ± 0.02	NS
Total protein	g/L	8	33.0 ± 0.83	31.7 ± 1.12	NS
		19	31.8 ± 1.49	31.6 ± 0.89	NS
Alkaline phosphatase	U/L	8	945 ± 72.4	968. ± 72.6	NS
		19	767 ± 68.4	1015 ± 53.6	NS
Alanine aminotransferase	U/L	8	22.1 ± 0.67	21.2 ± 0.85	NS
		19	31.7 ± 1.21	32.6 ± 1.20	NS
Aspartate aminotransferase	U/L	8	22.1 ± 1.67	23.5 ± 2.02	NS
		19	36.5 ± 7.17	32.7 ± 6.28	NS
Gamma-glutamyltransferase	U/L	8	26.2 ± 1.97	23.2 ± 2.19	NS
		19	16.4 ± 1.52	16.0 ± 1.21	NS
Total bilirubin	µmol/L	8	2.54 ± 0.53	3.00 ± 0.46	NS
		19	2.13 ± 0.27	2.68 ± 0.25	NS
Cholesterol	mmol/L	8	2.57 ± 0.18	2.28 ± 0.12	NS
		19	2.91 ± 0.17	3.06 ± 0.10	NS
Creatinine	µmol/L	8	50.5 ± 1.47	51.9 ± 3.41	NS
		19	46.7 ± 1.93	51.8 ± 1.99	NS
Creatine kinase	U/L	8	133 ± 23.4	108 ± 9.62	NS
		19	159 ± 32.1	147 ± 15.2	NS
Blood urea nitrogen	mmol/L	8	1.67 ± 0.31	2.29 ± 0.56	NS
		19	1.87 ± 0.28	1.38 ± 0.18	NS
Blood urea nitrogen/creatinine ratio		8	33.4 ± 6.35	46.6 ± 11.9	NS
		19	41.7 ± 7.28	27.7 ± 4.07	NS
Calcium	mmol/L	8	3.32 ± 0.08	3.11 ± 0.12	NS
		19	2.66 ± 0.11	2.70 ± 0.08	NS
Phosphate	mmol/L	8	2.01 ± 0.05	1.91 ± 0.05	NS

		19	2.29 ± 0.10	2.37 ± 0.05	NS
Magnesium	mmol/L	8	0.81 ± 0.02	0.81 ± 0.03	NS
		19	0.81 ± 0.04	0.87 ± 0.03	NS
Iron	μmol/L	8	6.84 ± 0.79	5.41 ± 0.57	NS
		19	9.03 ± 1.71	5.70 ± 0.93	NS
Sodium	mmol/L	8	144 ± 1.05	137 ± 5.85	NS
		19	140 ± 3.71	143 ± 2.78	NS
Potassium	mmol/L	8	5.33 ± 0.27	5.04 ± 0.29	NS
		19	4.32 ± 0.13	4.30 ± 0.09	NS

Values are expressed as mean ± SEM. Adjusted P-values are based on the Holm correction method. NS: Not significant. OPN, bovine milk osteopontin.

Supplementary Table S2: Full blood cytokine levels before and after LPS stimulation on day 8 and day 19 of preterm pigs fed a bovine milk diet (CON, n = 4-18) supplemented with OPN (OPN, n = 5-14).

		**Day 8**			**Day 19**		
**Parameter**	**Unit**	**OPN**	**CON**	**P-value**	**OPN**	**CON**	**P-value**
TNF-α Unstimulated	pg/mL	393 ± 383	338 ± 480	0.968			
TNF-α Stimulated	pg/mL	284 ± 301	144 ± 107	0.923	76.1 ± 37.0	69.2 ± 45.0	0.359
IL10 Unstimulated	pg/mL	456 ± 473	441 ± 268	0.924	169 ± 89	152 ± 43	0.473
IL10 Stimulated	pg/mL	705 ± 507	698 ± 343	0.472	1019 ± 703	836 ± 840	0.357
TNF-α ratio/IL10 ratio		1.16 ± 0.55	0.93 ± 0.49	0.487			

Data are expressed as mean ± SD. OPN, bovine milk osteopontin.
